# Comparison of the marginal adaptation of direct and indirect composite inlay restorations with optical coherence tomography

**DOI:** 10.1590/1678-775720160012

**Published:** 2016

**Authors:** Ayşe Gözde TÜRK, Metin SABUNCU, Sena ÜNAL, Banu ÖNAL, Mübin ULUSOY

**Affiliations:** 1 - Ege University, Faculty of Dentistry, Department of Prosthodontics, Izmir, Turkey.; 2 - Dokuz Eylül University, Department of Electrical and Electronics Engineering, Izmir, Turkey.; 3 - Ege University, Faculty of Dentistry, Department of Restorative Dentistry, Izmir, Turkey.

**Keywords:** Optical coherence tomography, Inlays, Dental marginal adaptation

## Abstract

**Objective:**

The purpose of the study was to use the photonic imaging modality of optical coherence tomography (OCT) to compare the marginal adaptation of composite inlays fabricated by direct and indirect techniques.

**Material and Methods:**

Class II cavities were prepared on 34 extracted human molar teeth. The cavities were randomly divided into two groups according to the inlay fabrication technique. The first group was directly restored on cavities with a composite (Esthet X HD, Dentsply, Germany) after isolating. The second group was indirectly restored with the same composite material. Marginal adaptations were scanned before cementation with an invisible infrared light beam of OCT (Thorlabs), allowing measurement in 200 µm intervals. Restorations were cemented with a self-adhesive cement resin (SmartCem2, Dentsply), and then marginal adaptations were again measured with OCT. Mean values were statistically compared by using independent-samples t-test and paired samples t-test (p<0.05), before and after cementation.

**Results:**

Direct inlays presented statistically smaller marginal discrepancy values than indirect inlays, before (p=0.00001442) and after (p=0.00001466) cementation. Marginal discrepancy values were increased for all restorations after cementation (p=0.00008839, p=0.000000952 for direct and indirect inlays, respectively). The mean marginal discrepancy value of the direct group increased from 56.88±20.04 µm to 91.88±31.7 µm, whereas the indirect group increased from 107.54±35.63 µm to 170.29±54.83 µm. Different techniques are available to detect marginal adaptation of restorations, but the OCT system can give quantitative information about resin cement thickness and its interaction between tooth and restoration in a nondestructive manner.

**Conclusions:**

Direct inlays presented smaller marginal discrepancy than indirect inlays. The marginal discrepancy values were increased for all restorations that refer to cement thickness after cementation.

## INTRODUCTION

During the last decade there has been an increasing demand for esthetic restorations in the posterior dentition. Esthetic restorations for Class II preparations include: direct composite restorations, direct composite inlays, indirect composites (inlays and onlays), ceramic inlays, and ceramic onlays^27^. Composites are limited for direct restoration of the larger stress-bearing posterior Class II cavities due to polymerization shrinkage effects and some limitations in mechanical properties^1^. Thermally post-cured composite inlays, however, are preferably recommended^1^. Ceramic materials are resistant to compressive forces, but they are susceptible to tensile stresses and more prone to fracture than composite materials^8^. It is stated that composite materials performed better stress distribution than ceramic materials in Class II cavities^1^. Composite inlays are usually chosen for the restoration of large defects^13^. Nowadays, many composite systems that can be used by both direct and indirect techniques are available. The direct composite inlay/onlay technique was introduced to improve the adaptation in Class II cavities^4^. In this technique, the composite is first light cured directly in the inlay cavity and then the inlay is removed from the cavity and post-cured. After the secondary cure, the inlay/onlay restoration is luted into place with composite luting materials^26^. In the indirect technique, an impression is taken after cavity preparation; then, it is sent to the laboratory to fabricate inlay restorations. The indirect technique improves the visual checking of marginal adaptation, proximal contacts, anatomic form, and polymerization shrinkage, compared with direct composite technique^26^. Directly fabricated inlays are less expensive, easily built up clinically, and demonstrate better marginal integrity than indirect ones^26^. Marginal adaptation, proximal contacts, and polymerization shrinkage can be also controlled with directly fabricated inlays rather than direct composite restorations.

The marginal adaptation is one of the important factors that determine the longevity of the restorations^25^. Insufficient sealing may lead to leakage of oral fluids along the interface between restorative material and tooth substrate, and can result in postoperative tooth sensitivity, marginal discoloration, and recurrent caries. Marginal adaptation of restorations has been evaluated by different methods such as sectioning the luted restorations and then measuring by optical microscope, scanning electron microscope (SEM), micro computed tomography (micro-CT), or transmission electron microscope (TEM) and replica technique. For SEM, sectioning of tooth/restorations involved is required to assess the presence of internal cracks and irregularities, which does not allow evaluating the marginal integrity *in vivo* ^23^. On the other hand, *in vivo* marginal integrity of restorations can be evaluated by SEM with the use of epoxy replicas^10^. Although the replica technique is a reliable and valid noninvasive method to determine the adaptation of restorations to tooth structure, deterioration of the silicone replica can occur, and defects in the area of measurement can affect the assessment of the film thickness with a microscope^17^. Recently, optical coherence tomography (OCT) was addressed as a noninvasive cross-sectional imaging of the internal biological system at the submicron scale^11^. It is a promising imaging modality, which does not require cutting and processing specimens and allows the visualization of microstructures of tissue and biomaterials in real time^3,11^.

The OCT was first used in dentistry in 1998, with *in vivo* imaging of hard and soft oral tissues^7^. It has since been used for evaluating marginal or internal adaptation of restorations^2,11,16,21-23,30^, crack or void evaluation in composites^24^, and enamel-ceramic interface^20^. Most studies have evaluated marginal adaptation of direct composite restorations with OCT^2,11,21-23^. According to our knowledge there has been few studies evaluating marginal adaptation of indirect restorations using OCT^16,30^ and no studies have compared the marginal integrity of direct and indirect composite restorations with OCT. The aim of this *in vitro* study was to quantitatively evaluate and compare the marginal adaptation of composite inlay restorations fabricated by direct and indirect techniques under OCT, and also compare the cement thickness of inlays after cementation. The null hypotheses tested were as follows: (1) there was no difference in marginal adaptation for the composite inlay fabrication techniques tested; (2) there were no changes in the marginal discrepancies of direct and indirect techniques after cementation.

## MATERIAL AND METHODS

### Specimen preparation

**Figure 1:**
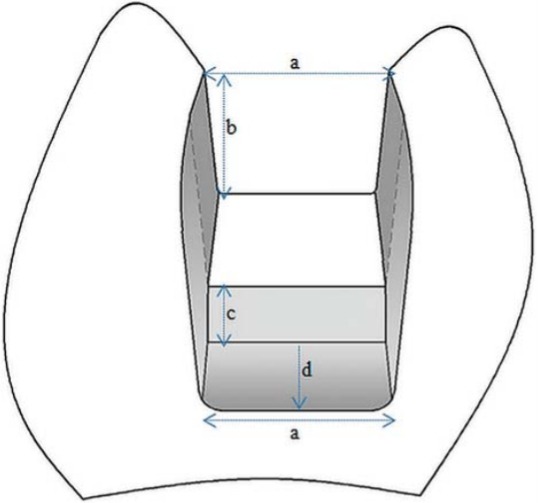
Schematic view of dimensions of Class II cavity (a=5 mm, b=4 mm, c=2 mm, d=2 mm)

After approval of the study protocol (Ege University, Medical Faculty, Ethics Committee no.: 14-12.1/12), 34 freshly extracted human first molar teeth, free of caries, cracks, and restorations were selected for the study. The teeth were approximately the same size and were stored in saline solution for up to 30 days. Teeth roots were embedded in plastic cylinders using a self-curing acrylic resin 3 mm away from the cervical line. The long axis of the tooth was oriented perpendicular to the surface of the acrylic block with a parallelometer (Degussa F1, DeguDent, Hanau, Germany). Class II cavities (Figure 1) were prepared by one operator. A 6° axial wall taper was obtained using the inlay cavity preparation bur (#959KR.018, Lot: 494511, Komet, Lemgo, Germany) by a high-speed air turbine under water-cooling. The internal angles were rounded and the enamel margins were not beveled. The cavities were rinsed with water and air-dried. Then, the teeth were randomly divided into two groups according to the inlay fabrication technique: direct and indirect groups (n=17).

**Figure 2:**
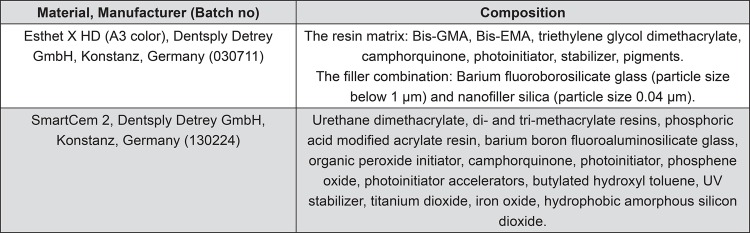
Composition, manufacturers, and batch numbers of the materials studied

In the direct group, two coats of glycerin were applied over all preparation walls and margins as a separating medium and left for three minutes for complete drying. A plastic matrix band (Omni-Matrix, Ultradent, South Jordan, UT, USA) was adapted. Afterwards, two consecutive 2 mm horizontal increments of Esthet X HD (Dentsply Detrey GmbH, Konstanz, Germany) (Figure 2) composite restorative material were applied to cavity walls and approximal contacts obtained by matrix band. The composite was anatomically shaped and each increment was light cured for 20 s with a LED-curing unit (Elipar S10, 3M ESPE, Seefeld, Germany) recommended by the manufacturer. The matrix band was removed and the inlay was cured for additional 20 s through the proximal, lingual, and buccal enamel walls. The inlay was carefully detached from the cavity with a fine probe and exposed to the post-curing for 2 minutes in a light-curing unit (Triad, Dentsply Trubyte, Canada). After all the inlays (n=17) were fabricated by one operator, they were checked for fit and adjusted with finishing burs under water cooling. The internal surfaces were gently sandblasted (50 µm alumina, 2 bar, 5 s).

In the indirect group, impressions were taken using prefabricated plastic caps with a radius of 10 mm as stock trays to reduce the bulk of the impression materials and a two-step technique (Affinis Precious, Coltane Whaledent, Switzerland) was used. The teeth were stored in distilled water at room temperature during the fabrication process. All impressions were stored at room temperature (25°C) for 1 hour before pouring to ensure a similar humidity effect on the setting of the impression material. Type IV dental die stone (GC Fujirock EP; GC Europe, Belgium) was mixed with a powder/water ratio of 100 g/20 mL under vacuum at 25 psi/Hg for 30 s and poured into the impressions according to the manufacturer’s instructions. After 1 hour, stone casts were separated from the impressions. One working cast and one master cast were obtained *per* inlay. One expert dental technician fabricated all indirect inlays. Two coats of glycerin were applied to all preparation walls on working cast and left for drying. Two horizontal increments of composite material (Esthet X HD) were applied and anatomically shaped. Each increment was light cured in light-curing unit (Triad) for 2 minutes. Before removing restoration from die, die was placed in Triad Unit for a final 2 minute curing according to the manufacturer’s instructions. Die stone was scraped away from the inlay margins to prevent accidental chipping of the restoration. Adjustments were made with finishing burs on master cast. Inlays were then checked in the respective cavities for marginal integrity using a silicone-disclosing medium (Fit Checker, GC-Germany, Munich, Germany). Internal surfaces were sandblasted (50 µm alumina, 2 bar, 5 s).

### OCT analysis

**Figure 3:**
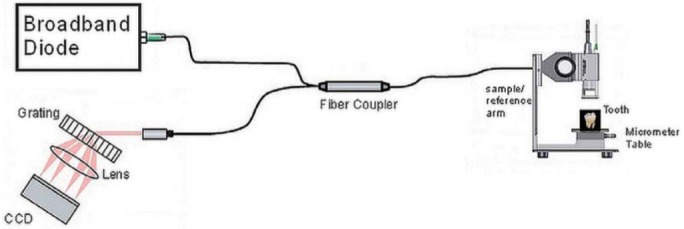
The spectral domain optical coherence tomography (OCT) setup. The broadband diode light source is a super luminescent diode with central wavelength 930 nm and bandwidth 100 nm. A fiber coupler combines the light waves reflected off the sample and reference arms. The interference signal is then separated into its frequency components through an optical grating and sent to a charge-coupled device (CCD). The tooth is placed on a table in the sample arm

**Figure 4:**
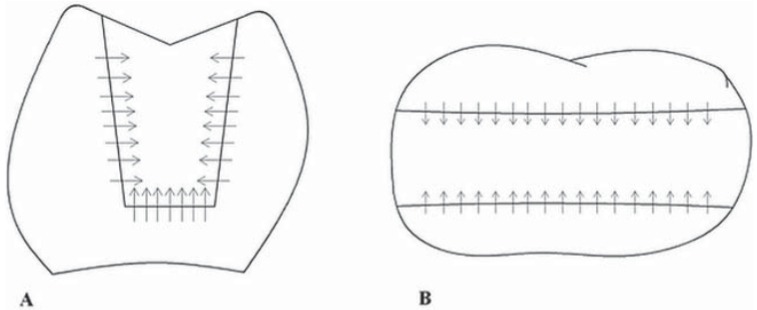
Pictorial representation of the measurement procedure. Arrows represent the optical coherence tomography (OCT) beam scans. The infrared beam was scanned over tooth-marginal discrepancy (air or resin cement)-inlay surfaces. After each scan the beam was moved 200 µm and the scan was repeated. A) For the mesial and distal surfaces, buccal to inlay, gingival to inlay, and lingual to inlay measurements were performed. B) For the occlusal surface, buccal to inlay and lingual to inlay measurements were carried out

In order to determine marginal discrepancies of the inlay restorations, all 34 teeth were measured by OCT before cementation as a first step. The measurement procedure that reveals the marginal discrepancy was as follows: the tooth was placed in the sample arm and the OCT diode was focused on the tooth, as shown in Figure 3. It was necessary to fix the length of the arm by monitoring the signal reflected of the tooth-air interface. The reference arm length was tuned until a sharp image was seen on the CCD camera, a camera incorporating a charge-coupled device, enabling a coarse adjustment. Then, by optimizing the OCT spectrum on the B-scan, the fine adjustment was accomplished. After this step, the infrared beam was scanned over the tooth until distance between tooth and inlay restoration (which corresponds to the discontinuity caused by air before cementation or resin cement after cementation) was detected. The tooth was placed in the reference arm in such a way that the light beam first hit the tooth from the top. The light beam was orthogonally scanned to the tooth-inlay interface in such a way that the infrared beam traversed over the tooth surface, air (distance between tooth-inlay), and inlay restoration regions sequentially. The entire tooth-restoration margins were scanned, which means from one approximal surface to the other including the cavosurface margins. The infrared beam was scanned over tooth-air-inlay surfaces. After each scan the beam was moved 200 μm and the scan was repeated. For the mesial and distal surfaces, buccal to inlay, gingival to inlay, and lingual to inlay measurements were performed. For the occlusal surface, buccal to inlay and lingual to inlay measurements were carried out (Figure 4).

The system took spectral domain OCT images. The A-scan Line rate was 1.2 kHz and B-scan frame rate was at a 512 line/frame. The resolution of OCT images in depth and lateral scan were 7 µm and 8 µm respectively. The imaging depth was around 1.7 mm and the measurements had a Signal-to-Noise Ratio (SNR) of 83 dB.

**Figure 5:**
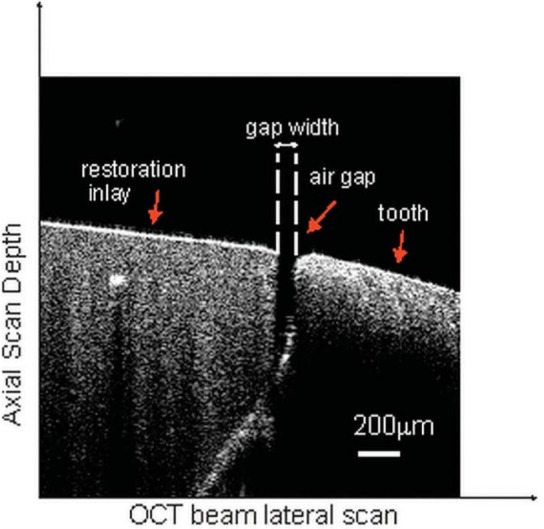
A representative optical coherence tomography (OCT) scan of a tooth from the direct group before cementation. The gap width represents the marginal discrepancy that was measured from the air gap between the tooth and inlay restoration

**Figure 6:**
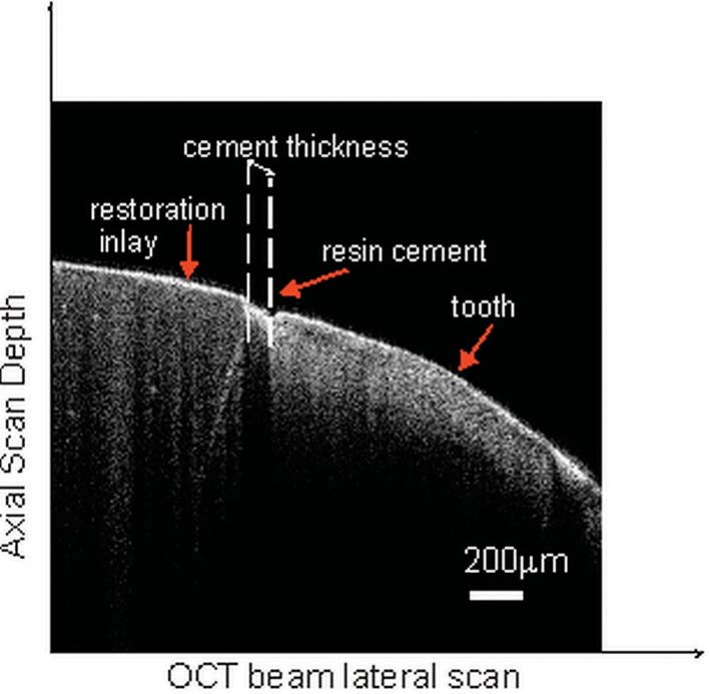
Optical coherence tomography (OCT) scan of a tooth from the direct group after cementation. The marginal discrepancy was measured from the resin cement thickness

On each margin, OCT measurements were obtained in 200 µm intervals, which resulted in 140 OCT scans on average per tooth. The table on which the tooth was placed was adjustable with a micrometer screw to achieve a smooth and precise 200 µm incremental shift between successive measurements. This allowed for taking OCT B-scans and thus measuring the marginal gap in 200 µm intervals. Using the ruler tool on the image processing software, the marginal discrepancy (corresponding to air before cementation and resin thickness after cementation, as shown in Figures 5 and 6) was measured and recorded during each scan. These data were then averaged to estimate mean marginal discrepancy values. The same measurement procedure was applied to all 34 teeth after the cementation process.

### Cementation

All preparation walls were cleaned with pumice, and inlay restorations were ultrasonically cleaned in distilled water for 10 min and then air-dried. Inlays were luted with self-adhesive dual-cure resin cement (SmartCem2, Dentsply, Konstanz, Germany) (Figure 2). The cement was mixed with the manufacturer’s dispenser and applied to the cavity walls. Then, the restoration was positioned into the cavity by using a custom-made tip in a universal machine (Shimadzu, Tokyo, Japan) for standardization of cementation procedures. Excess cement was removed, then light curing (Elipar) was applied for 20 s through the proximal, lingual, buccal, and occlusal enamel walls recommended by the manufacturer, and kept under 50 N of static load for 6 min (total curing time). Finishing was performed with finishing diamonds (Prisma) and polishing disks (SofLex Pop-on, 3M ESPE, St Paul, USA).

### Statistical analysis

Marginal discrepancy values were compared by statistical parametric tests using SPSS 13.0 (SPSS Corporation, Chicago, IL, USA). Differences between direct and indirect technique were evaluated by independent-samples t-test. After a homogeneity of variance test was performed using Levene test, before and after cementation data were compared by paired samples t-test at a significance level of alpha=0.05.

## RESULTS

The OCT scans taken before (Figure 5) and after (Figure 6) cementation enabled to observe and measure marginal discrepancies of the inlay restorations. The resin cement thickness, its structure, and interaction between the tooth and the restoration were also observed and measured.

After assessing all the data statistically, mean marginal discrepancy values were calculated as summarized in Table 1. There were statistically significant differences between the marginal discrepancy values of direct and indirect groups. The direct group showed smaller values than the indirect group before (p=0.00001442) and after (p=0.00001466) cementation. After cementation, marginal discrepancy values increased significantly both for the direct (p=0.00008839) and indirect (p=0.000000952) groups. The percentage of increase was 61.53% for the direct group and 58.35% for the indirect group.

**Table 1:** Mean standard deviation (SD) and minimum and maximum marginal discrepancy values (µm) of direct and indirect inlays before and after cementation (n=17)

Groups	Mean	SD	Minimum	Maximum
Before				
Direct group	56.88^A^	20.00	33.38	116.82
Indirect group	107.54^B^	35.63	60.07	188.23
After				
Direct group	91.88^C^	31.00	65.09	176.35
Indirect group	170.29^D^	54.83	96.44	288.39

Different superscript letters represent statistically significant differences among groups [independent-samples t-test, paired samples t-test (p<0.05)]

## DISCUSSION

Optical Coherence Tomography, introduced in 1991, is a powerful tool that produces non-contact, noninvasive tomographic images of biological tissues^12^. The OCT images are obtained by measuring the echo time delay and intensity of backscattered light from a specimen. Such tool uses inherent differences in the index of refraction in tissue rather than enhancement with dyes to differentiate tissue types^20^. Because OCT is a non-contact and nondestructive method, it was useful in taking sensitive measurements in different areas such as ophthalmology and cardiology^5^. The OCT was first used in dentistry in 1998, with *in vivo* imaging of oral tissues^7^. Marginal or internal adaptations of composites have been evaluated by OCT either qualitatively^23^ or quantitatively^11,21,22,30^. In these studies, interfacial gaps were confirmed by using a confocal laser scanning microscope (CLSM) or optical microscope. In addition, other studies stated that the OCT system can be employed as a quantitative and complementary tool for analyzing the fracture propagation, defects, and gaps^15,20^. In the present study, the photonic imaging modality of OCT was utilized to quantitatively compare the marginal adaptation of composite inlay restorations fabricated by direct and indirect techniques. Some studies used silver penetration into the interfacial gap that behaved as a metallic contrast agent, which enhanced the OCT reflection in their study^11,21^. On the other hand, contrast agents were not used in other OCT studies regarding the examination of dental restorations^2,16,20,22,23,30^. In the present study, the marginal adaptations of direct and indirect inlays were qualitatively, quantitatively, and noninvasively compared by OCT without a contrast agent, and a large cavity scheme was used to represent the clinical situation.

In previous OCT studies, different wavelengths, such as 800 nm^22^, 830-1280 nm^7^, 930 nm^18,23^, 1260-1360 nm^11,15^, 1310 nm^19,30^, and 1319 nm^21^, have been used. The studies, which have used 1260 nm and more wavelength source with spatial resolutions of 10 µm, have evaluated the resin-dentin interface of the cavity floor, defects of the composites, and internal adaptation or sealing performance of the resin cements. It is stated that when measuring subsurface structures, such as micro leakages or demineralized areas, it would be advantageous to have a higher wavelength source of 1310 nm or more because lower wavelength light penetrates less in tissue^6^. In our study, 930 nm wavelength of OCT system (Thorlabs) with spatial resolution of 7 µm and a bandwidth of 100 nm was used to measure the marginal discrepancies between teeth and inlay. Since the imaging depth of OCT system used in this study is 1.7 mm, the internal adaptation of inlays could not be detected due to the 4 mm cavity depth. Similar to the studies that have used 930 nm, the measurement locations were on the surface of the tooth^18^, not on inner surfaces, and marginal analysis^23^ was performed. According to the results of this study, the null hypothesis 1 was rejected because direct inlay technique used in this study seems to have clinically acceptable marginal discrepancy value than indirect one. Fabrication stages of indirect composite inlays, including impression and the die production steps, could explain greater marginal discrepancy values in indirect composite inlays. Marginal discrepancy values for direct and indirect inlays were found to be 91.88 µm and 170.29 µm respectively. Marginal discrepancy values ranging from 48 to 219 µm have been reported for various indirect composite and ceramic inlays^9,14,29^. However, it is recommended that marginal adaptation of inlays should be less than 100 µm^10^. Although inadequate adaptation of inlays can be compensated by resin luting cement at the margins of a restoration, it has been shown that an accurately fitting restoration is prerequisite for long-term success. It is stated that significant resin luting cement wear was also observed around wide marginal adaptation values (>150 µm), and a good marginal adaptation would significantly reduce the wear of resin luting cements in clinical conditions^10^.

In addition, in the present study the OCT images showed information about the resin cement thickness, its structure, and interaction between the tooth and the restoration. Self-adhesive resin cement was used for cementation to eliminate the operator factor of etch-and-rinse multi step adhesives. After analyzing the measurements, we found that marginal discrepancy values of direct and indirect inlays were increased 35 µm and 62.75 µm, respectively, after cementation. Thus, the null hypothesis 2 was rejected. The increase in the discrepancy value can be related to the resin cement viscosity or problematic discharge of excess cement because of the complex cavity geometry. This result was supported by other studies, which found a significant increase in the marginal discrepancy values after cementation^28^, which was attributed to the increase of hydraulic pressure of the resin luting cement^28^.

Other imaging methods, such as SEM, micro-CT, or optical microscope, could compare marginal adaptation of inlays. For SEM, the specimens should be covered by gold for electrical current discharge. This may not be appropriate for comparing the before and after cementation marginal discrepancy values. The gold sputtered layer, required for SEM images, can mask details that OCT images do not encounter^23^. Compared with the optical microscope, the OCT system penetrates the sample up to a depth of 2-3 mm^22^, showing its internal structure with high resolution. Micro-CT can also provide high-resolution tomographic images such as the OCT system. The main advantage of OCT over micro-CT system and which makes OCT suitable in the biomedical sector is the absence of toxic effects such as ionizing radiation.

Clinical OCT systems are now becoming an effective, nondestructive, and suitable method for evaluating marginal adaptations of restorations with the development of small, flexible fiber optic OCT probes that can easily access the oral cavity. There are studies evaluating restorations *in vivo* ^7,15,19^ and further studies should be performed.

## CONCLUSION

Within the limitations of this *in vitro* study, marginal discrepancies of inlay restorations were quantitatively and noninvasively evaluated by the OCT system. The following conclusions may be drawn: direct inlays presented smaller marginal gap values than indirect inlays. The marginal gap values were increased for all restorations after cementation.
